# Intrinsic Functional Connectivity Networks in Healthy Elderly Subjects: A Multiparametric Approach with Structural Connectivity Analysis

**DOI:** 10.1155/2014/947252

**Published:** 2014-05-29

**Authors:** Martin Gorges, Hans-Peter Müller, Albert C. Ludolph, Volker Rasche, Jan Kassubek

**Affiliations:** ^1^Department of Neurology, University of Ulm, Oberer Eselsberg 45, 89081 Ulm, Germany; ^2^Experimental Cardiovascular Imaging, Core Facility Small Animal MRI, University of Ulm, Albert-Einstein-Allee 23, 89081 Ulm, Germany

## Abstract

Intrinsic functional connectivity magnetic resonance imaging (iFCMRI) provides an encouraging approach for mapping large-scale intrinsic connectivity networks (ICNs) in the “resting” brain. Structural connections as measured by diffusion tensor imaging (DTI) are a major constraint on the identified ICNs. This study aimed at the combined investigation of ten well-defined ICNs in healthy elderly subjects at single subject level as well as at the group level, together with the underlying structural connectivity. IFCMRI and DTI data were acquired in twelve subjects (68 ± 7 years) at a 3T scanner and were studied using the *tensor imaging and fiber tracking* software package. The seed-based iFCMRI analysis approach was comprehensively performed with DTI analysis, following standardized procedures including an 8-step processing of iFCMRI data. Our findings demonstrated robust ICNs at the single subject level and conclusive brain maps at the group level in the healthy elderly sample, supported by the complementary fiber tractography. The findings demonstrated here provide a methodological framework for future comparisons of pathological (e.g., neurodegenerative) conditions with healthy controls on the basis of multiparametric functional connectivity mapping.

## 1. Introduction

Soon after the development of functional MRI [1], Biswal et al. described oscillatory hemodynamics in low frequency range (<0.1 Hz) within regions in the motor cortex by demonstrating spontaneous blood oxygenation level dependent (BOLD) fluctuations in a highly correlated manner between functionally associated brain areas when the brain is “at rest” [2]. Mapping the functional connectome of the human brain remains challenging [3], since intrinsic functional connectivity (iFC) analysis in the “resting-state” is only an indirect proxy of the “ongoing” brain's hemodynamics. Besides the fact that the acquired data might be confounded by several factors such as respiratory, pulsatile, or cardiovascular artifacts [4, 5], shortcomings of postprocessing are assumed to influence the shape of the BOLD response [6]. There is growing awareness of a large scale functional brain architecture structured in a topological manner [7] and attributed to specific functional explication [8]. Notably, the functionally interacting portions of the brain that were demonstrated in the absence of specific tasks (“task-free”) correspond to a large extent to those regions that reveal “task-induced” coactivations in task-based functional MRI [9]. Neural signaling between brain areas is markedly constrained by the brain's anatomy and hence by axonal bundles (fibers) that interconnect different brain segments/regions [10], forming an efficient network and comprising interconnected hubs [11].

The probably most extensively studied intrinsic connectivity network (ICN) is the default mode network (DMN) [12–14] that has been initially described by Raichle et al. [15]. This large scale system includes areas revealing the highest coherent low frequency oscillations in the absence of a specific task [12]. In the past decade many ICNs have emerged consistently identified by means of iFCMRI comprising the dorsal and ventral attention systems [16, 17], executive control and salience processing [18], primary visual and visual associative systems [8, 19], cerebellar network [8, 20], functional integrity of the cingulate cortex [21], and further networks [20]. These ICNs were identified using the “hypothesis-driven” seed-based approach. Additionally, “data-driven” independent component analysis exists which allow for the identification of a series of independent components that could be interpreted as an extract of distinct functionally organized networks [4, 19, 22].

A number of studies investigated the relationship between functional and structural connectivity (see e.g., [23] for a review), showing convergence between the strengths of resting-state functional connectivity and structural connectivity. Since it is commonly assumed that functional connectivity reflects structural connectivity in the brain [23–25], brain connectivity has been proven to play an essential role in the pathological state [12, 26–28] as well as in healthy aging where changes in functional [29] and structural connectivity [30] have been reported during the lifespan. Still, different connectivity measures as used in multiparametric approaches (e.g., BOLD fMRI and DTI measures) are a promising issue for further research and clinical applications.

This study aimed at the investigation of ten well-defined ICNs as functional brain maps in healthy elderly subjects together with the complementary analysis of the underlying structural connectivity. From the methodological view, the functional and structural connectivity analysis was performed by use of the in-house developed software package* tensor imaging and fiber tracking* (TIFT) [31, 32] that is well-established for diffusion tensor imaging (DTI) analysis procedures [31]. For the analysis of the iFCMRI data, a standardized data processing approach will be demonstrated on an item-by-item basis. We utilized a single-voxel seed-based approach in order to identify the ICNs at single subject level with subsequent group level computation of brain maps from individuals' data. This way, we aimed at demonstrating our concept to combine iFCMRI with DTI data on the basis of recent studies [10]. These algorithms used in the TIFT software were evaluated in a group of healthy elderly. The investigation of the elderly adult has major impact when defining a control group for assessments in many studies in aged individuals such as in neurodegenerative conditions that commonly manifest in late life. Thus, a reference compilation of ICNs together with the underlying anatomical structure is of great interest for clinical comparisons.

## 2. Materials and Methods

### 2.1. Subjects

All subjects gave written informed consent for the MRI protocol according to institutional guidelines. The study had been approved by the Ethics Committee of the University of Ulm and was performed in accordance with the ethical standards laid down in the 1964 Declaration of Helsinki and its later amendments.

Twelve healthy volunteers (mean age 68 ± 7 years, M/F ratio 7/5) were recruited from the controls' database of the Department of Neurology, University of Ulm. Detailed demographic data are listed in Table 1. The investigated group consisted of volunteers with higher education without any psychiatric disorders or history of neurological or other medical conditions and free of any cognitive problems. Overall cognitive performance was screened by mini-mental state examination (MMSE) [33] as well as by global dementia screening (DemTect) [34]. Exclusion criteria were cerebrovascular diseases, psychiatric abnormalities, severe hearing damage, or significant white matter lesion load such as periventricular or deep white matter hyperintensities [35].

### 2.2. MRI Acquisition

MRI scanning was performed at a 3-Tesla clinical scanner (Magnetom Allegra, Siemens, Erlangen, Germany; SyngoMRA30). The protocol included a “resting-state” iFCMRI sequence, a DTI sequence, and a T1-weighted 3D magnetization-prepared gradient echo sequence (MPRAGE). The iFCMRI scanning protocol consisted of 200 volumes (36 slices, 64 × 64 pixels, slice thickness 3.5 mm, and pixel size 3.5 mm × 3.5 mm). The echo time (TE) and repetition time (TR) were 30 ms and 2200 ms, respectively. All participants were instructed to keep relaxed and motionless with their eyes closed but awake in the absence of goal-directed attention during iFCMRI data acquisition. The DTI protocol consisted of 31 gradient directions (GD), including one *b* = 0 reference (72 slices, 128 × 128 pixels). The slice thickness was 2.0 mm; in-plane pixel size was 2.0 mm × 2.0 mm. TE and TR were 95 ms and 12700 ms; *b* was 1000 s/mm^2^. For the morphological background, a T1-weighted MPRAGE was recorded for each control subject (TR 2500 ms, TE 4.32 ms, matrix size 256 × 256 pixels, 192 slices, slice thickness 1.0 mm, and in-plane pixel size 1.0 × 1.0 mm^2^).

### 2.3. iFCMRI Data Preprocessing: Overview

All described algorithms and postprocessing were integrated in the previously described analysis software TIFT. A standardized preprocessing procedure was applied to all iFCMRI data, comprising the following components.

#### 2.3.1. Preprocessing Step  1: Quality Control and Motion Correction

To insure sufficient image quality, all volumes of the EPI and MPRAGE images were visually inspected for proper registration. As head motion influences iFCMRI [36], motion corrected data were obtained from the scanner software (syngo MRVA30A, Siemens, Erlangen, Germany) that provides a 3-dimensional elastic motion correction in all directions.

#### 2.3.2. Preprocessing Step  2: Resampling on a Cubic 1 mm Grid

The data were resampled from 3.5 × 3.5 × 3.5 mm^3^ to a cubic 1 mm grid of a 256 × 256 × 256 matrix by means of a nonparametric *k*-nearest neighbor regression approach [37] using the average voxel intensity of the *k*-nearest neighbor voxels weighted by the inverse of their distance. Upsampling to a cubic 1 mm grid has already been performed in previous studies (e.g., [31]). The advantage of using the identical voxel resolution for multiparametric studies such as this bimodal approach (DTI, iFCMRI) is that in this way voxel locations could be easily and unambiguously transferred from one modality to another and vice versa. In addition, performing analysis in a cubic 1 mm grid provides a prerequisite for the utilized seed-voxel approach.

#### 2.3.3. Preprocessing Step  3: Stereotaxic Normalization

**Figure 1:**
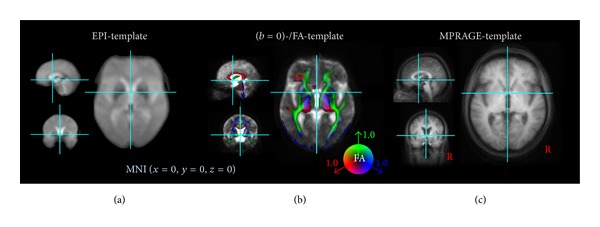
Templates in MNI stereotaxic space used for this study. (a) EPI template. (b) DTI templates ((*b* = 0)-template and overlaid color-coded fractional anisotropy- (FA-) template), FA threshold 0.2. (c) MPRAGE template. Each template was computed for healthy elderly (*N* = 12) included in this study with display focus at the anterior commissure.

Normalization of the rescaled EPI images (1.0 × 1.0 × 1.0 mm^3^) to the Montreal Neurological Institute (MNI) stereotaxic standard space [38] was accomplished [32]. First eight landmarks were defined in the EPI data in the first volume for each subject. A linear transformation in all 6 degrees of freedom (*x*, *y*, *z*, pitch, roll, and yaw) into MNI space was performed according to the coordinates of these landmarks. This procedure was applied separately for each MRI modality (iFCMRI, DTI, MPRAGE) for all subjects' data (first volume) included in this study in order to compute a modality specific template by arithmetically averaging the voxel intensities of all individual MNI transformed images. The deformation procedure was refined in the second iteration step by nonlinear normalization of the individual EPI data onto the study-specific template following the basic ideas of Ashburner and Friston [39] of minimizing the squared differences of regional intensities between the individual first EPI volume and the EPI template. Validation of the MNI normalization was performed by calculating Pearson's product correlation coefficient between each individual EPI image (first volume of data series) and the EPI template as a quantitative measure. The landmarks were refined for each subject whose normalization was unacceptable according to a correlation coefficient of *r* < 0.8. The same template-based normalization procedure was applied to the high-resolution MPRAGE images used for seed-voxel definition and for the display of results on a morphological background.  Figure 1 displays templates for the different modalities in comparison to the MPRAGE template.

#### 2.3.4. Preprocessing Step  4: Spatial Filtering

**Figure 2:**
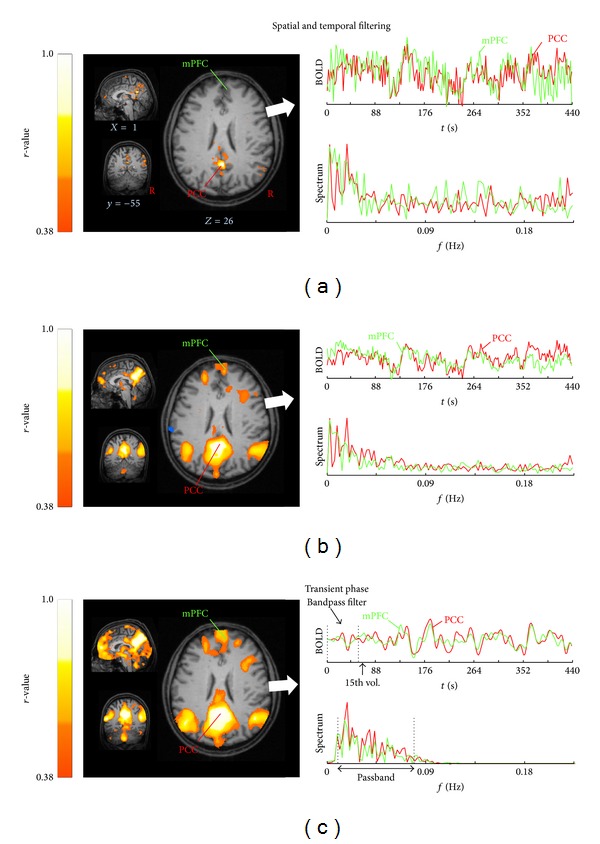
The effect of spatial and temporal filtering exemplified for one representative subject. Most informative orthogonal slices (left column) in the MNI stereotaxic space show the default mode network (DMN) computed for a seed-voxel in posterior cingulate cortex (PCC,* xyz*, 0−5526) for (a) motion corrected and resampled data to 1 mm cubic grid, (b) after spatial smoothing with a 3D-Gaussian kernel (8 mm FWHM), and (c) after temporal linear detrending and bandpass filtering (0.01–0.08 Hz). Time rows with corresponding frequency spectrum (right column) for the seed-voxel (red trace) and a voxel-time course extracted from the medial prefrontal cortex (mPFC, green trace,* x y z*, 1/50/22). Connectivity strengths are shown in hot colors, thresholded for |*r* | >0.38 corresponding to *P* < 0.0000001 and overlaid on the individual MPRAGE (1.0 × 1.0 × 1.0 mm^3^).

Spatial filtering was applied to the EPI series of each subject's data by using a 7 mm full-width at half maximum (FWHM) Gaussian blur filter (3-dimensional bell shape representing normal distribution). The filter width of 7 mm equals twice the recording voxel size of 3.5 mm which is a common choice according to the assumption that the Gaussian filter is designed as a “matched filter” [40]. The matched filter theorem states that the width of the filter used to process the data should be tailored to the size of the structure under investigation [41]. None of the data sets for both modalities iFCMRI and DTI had to be excluded prior to the analysis due to unacceptable artefacts. The effect of spatial filtering is exemplified in Figure 2.

#### 2.3.5. Preprocessing Step  5: Temporal Linear Detrending and Temporal Bandpass Filtering

Possible scanner drifts during the iFCMRI data acquisition were voxelwise removed over each volume by linear detrending [42]. Linear detrending was performed by subtracting the linear fit of the voxel time course. The time courses were further bandpass-filtered since iFCMRI data analysis is based on the coherence of low-frequency BOLD fluctuations. The frequency spectrum was band-limited for cutoff frequencies in the range of 0.01 < *f* < 0.08 Hz [4, 6, 43] using a 6th-order Butterworth bandpass filter design. The first 15 out of 200 volumes of each time course were discarded due to the transient filter response (see Figure 2(c)) and due to scanner oscillations at the beginning of iFCMRI data acquisition. Moreover, this commonly applied procedure allows the participant to adapt to the experimental condition [44].

#### 2.3.6. Processing Step  6: Seed-Based Correlation

**Figure 3:**
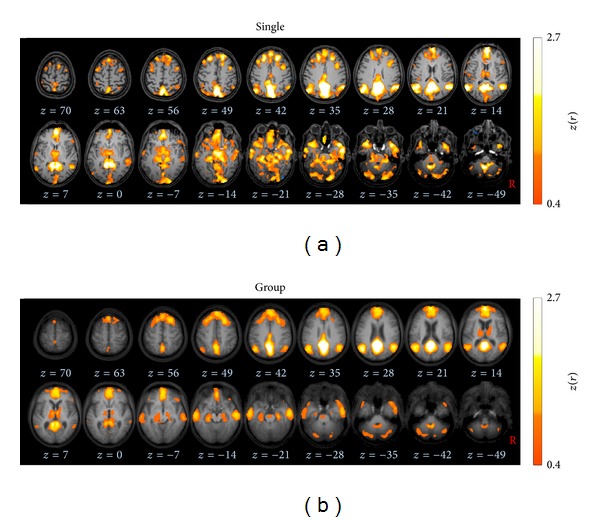
Axial slices in the MNI stereotaxic space show the default mode network (DMN) computed for a seed-voxel in posterior cingulate cortex (PCC,* xyz*, 0−5526). Connectivity strengths are depicted in hot colors, thresholded for |*z*(*r*)|>0.4 corresponding to *P* < 0.0000001. (a) Results for a representative subject (male, 60 years) overlaid on the individual MPRAGE images. (b) Group averaged brain map for all subjects (*N* = 12) displayed on the averaged MPRAGE background.

**Figure 4:**
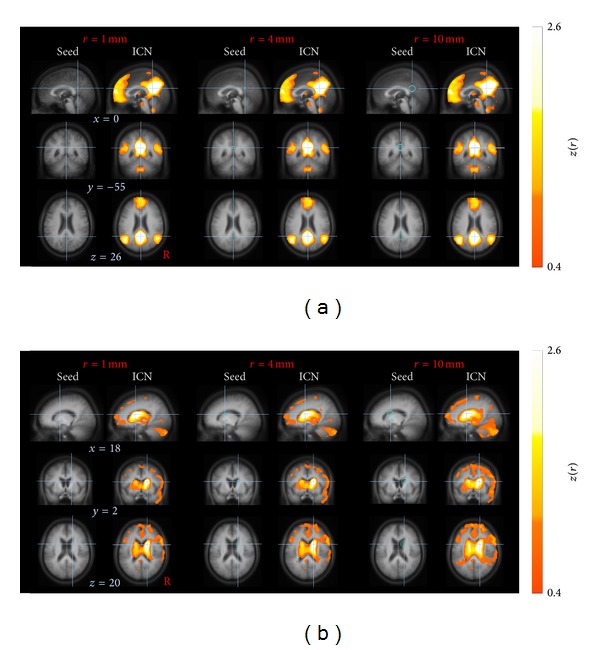
Effect of voxel-seed versus spherical ROI seeds at group level. (a) Default mode network (DMN) computed for a posterior cingulated cortex (PCC) seed. (b) Basal ganglia thalamic (BT) ICN computed for a caudate seed. Crosshairs on orthogonal slices indicate the voxel-seed location (left column), the center of the spherical ROI with 4 mm radius (center) and 10 mm radius (right). Data are shown in 1 mm cubic grid in the MNI space on the averaged MPRAGE image. The computation of the ICNs (right column of each pair) revealed similar results for a seed-voxel compared to a spherical ROI for the PCC seed (a). However, for smaller structures such as the caudate, the resulting brain map pointed towards a slightly different and more diffuse pattern with spherical radii larger than 4 mm (b).

Large scale correlation maps were computed according to the seed-based approach [2] in accordance with recent studies [7, 45, 46]. Ten well-defined ICNs [8, 20, 27] were computed by placing seed-voxels (i.e., encompassing one voxel only) into regions that had been consistently reported to serve as central hubs for the respective ICN as listed in Table 2. The exact location of the seed was manually refined based on the subjects' averaged high-resolution T1-images (MPRAGE) according to the standardized MNI atlas [38].  Figure 3 displays the DMN calculated for a representative single subject in the MNI space. As a novel aspect, the time series of one single voxel (i.e., the seed-voxel) was extracted in contrast to common approaches such as averaging the time series of voxels within a defined seed region [44, 47, 48] or taking each of the voxels within the given ROI as a seed-voxel [45]. The extracted time course of the seed-voxel or the averaged time course extracted from all voxels within the ROI spheres was correlated with the time series of all other voxels across the whole brain, yielding a corresponding correlation coefficient (*r*-value) for each voxel. The similarity of using a seed-voxel (1.0 × 1.0 × 1.0 mm^3^) or a spherical ROI with a radius of about 4 mm is shown in Figure 4. Nevertheless, using ROIs larger than 4 mm, the resulting brain maps slightly differ for “anatomically small” seed regions as exemplified for a 10 mm spherical ROI radius placed in the caudate in Figure 4(b) (right panel). Since the time courses were considered to be normally distributed, correlations were computed by use of the parametric Pearson's product moment correlation method.

#### 2.3.7. Processing Step  7: *z*(*r*) Transformation

Fisher's *r*- to *z*-transformation [54] was applied voxelwise to improve the normal distribution of the individual's connectivity maps as *Z* statistic images [55]. Each voxel corresponds to a *z*(*r*) score representing its connectivity strengths with respect to the seed-voxel of the respective ICN. As an example, Figure 3 displays the default mode network (DMN) calculated for a representative single subject on a cubic 1 mm grid in MNI coordinates.

#### 2.3.8. Processing Step  8: Averaging Individual Brain Maps

**Figure 5:**
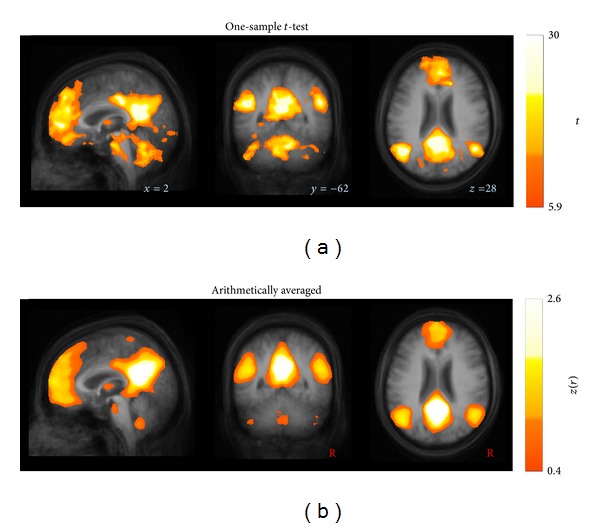
Similarity of ICNs at group level computed by (a) a one-sample *t*-test compared to (b) arithmetically averaged, as exemplified for the most informative orthogonal slices depicting the default mode network. The two-sided one-sample *t*-test was thresholded at *P* < 0.001, corrected for multiple comparisons using the false discovery rate. Data are shown in stereotaxic MNI space in a 1 mm^3^ cubic grid.

In order to obtain the ICN at the group level, the *z*(*r*) scores were arithmetically averaged voxelwise [45, 46, 49]. The brain maps at group level were statistically validated by also applying a two-sided one-sample *t*-test [44]. Arithmetical averaging and the application of one-sample *t*-test revealed similar results (Figure 5).

### 2.4. DTI Data Processing

The DTI analysis software TIFT was used for DTI data processing. In order to perform a spatial normalization in the MNI stereotaxic standard space, a study-specific (*b* = 0)-template and a fractional anisotropy- (FA-) template (see Figure 1(b)) had to be created [31]. As the nonaffine registration to an FA-template has the advantage of providing more contrast in comparison to (*b* = 0)-images [56], a FA-template was defined by averaging all individually derived FA maps for the healthy elderly.

Prior to averaging, DTI data were controlled for motion corrupted volumes by a recently described technique [57]; it was found that no volumes had to be excluded for further analysis. The subsequent averaging procedure requires careful treatment of the orientational information which is preserved during the normalization process [32, 58]. After this normalization procedure, all individual DTI data sets were used for the calculation of the second-rank diffusion tensor and the FA for quantification of the diffusion anisotropy, according to standard methods [59]. In order to apply group based fiber tracking (FT) algorithms, averaged DTI data sets were calculated from all subjects' data sets by arithmetic averaging of the MNI transformed data. In this manner, an averaged DTI data set was calculated while preserving directional information of individual data sets (for details, see [32, 60]). These averaged DTI data sets were then used to identify pathways for defined brain structures. Tractography was performed by using a streamline tracking technique. Manually defined seed points were the basis for the consecutive FT [31]. In order to improve FT performance, additional control data sets with 49 GD were included in the study. Parameters for FT represented a dot product threshold between two FT steps of 0.9, a FA threshold of 0.2, and a seed-voxel radius of 5 mm. Seeds for the FT corresponding to the ICNs are listed in Table 3.

## 3. Results

**Figure 6:**
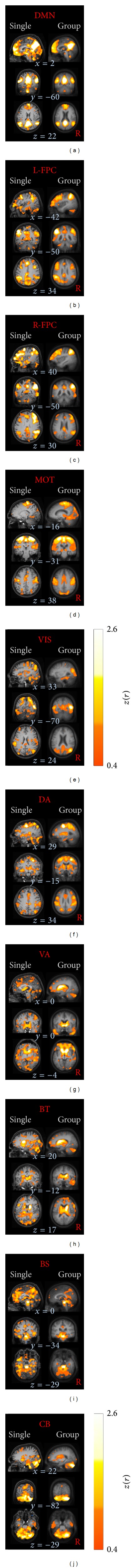
Orthogonal slices of ten well-matched pairs of ICNs ((a)–(j)) in the stereotaxic MNI space for a representative single subject (male, 60 years) overlaid on the individual MPRAGE (left columns) and for the complete healthy subject group (*N* = 12) displayed on their averaged MPRAGE (right columns). Connectivity strengths of brain maps are depicted in hot colors, thresholded for |*z*(*r*)|>0.4 corresponding to *P* < 0.0000001. (a) The default mode ICN (DMN). ((b), (c)) The left and right frontoparietal control (FPC) ICNs. (d) The motor (MOT) ICN. (e) The visuospatial (VIS) ICN. (f) The dorsal attention (DA) system. (g) The ventral attention (VA) ICN. (h) The basal ganglia thalamic (BT) ICN. (i) The brainstem (BS) ICN. (j) The cerebellar (CB) ICN.

Ten well-described ICNs were unambiguously identified by using seed-voxels placed into regions as listed in Table 2. In order to illustrate the performance of averaging, a representative single subject DMN was juxtaposed to the group-averaged DMN in Figure 3. More specifically, Figure 6 illustrates ten identified single subject ICNs in juxtaposition to the ten group-averaged ICNs according to Table 2 as follows.

(a) The* default mode network* (DMN) (Figure 6(a)) was identified by seeding the PCC with the adjacent precuneus region. The brain map covers the medial parietal cortex comprising bilateral temporal areas around their midline extending to inferior parietal regions. In addition, the medial frontal cortex reveals activity in ventromedial, anteriomedial, and dorsomedial areas, the frontal pole, and the anterior cingulate. Weaker activity was observed in the bilateral hippocampal formation and parts of dorsolateral prefrontal cortex.

(b, c) Figures 6(b) and 6(c) show the* left* and* right frontoparietal control *ICNs yielded by seeds in the left and right middle temporal area, respectively. Within these spatial maps, activity was observed in several frontoparietal areas comprising the dorsolateral prefrontal cortex, frontal pole as well as lateral occipital area, inferior parietal cortex, and parts of the posterior cingulate cortex. As a relay between cortical and subcortical areas, also bilateral thalamic activation was found.

(d) The* motor* ICN (Figure 6(d)) was computed for a seed region within the left motor cortex revealing similar activation in both hemispheres comprising the sensory-motor and motor association systems. These included pre- and postcentral motor regions and the supplementary motor area. Moreover, weaker activation was found in the visual association areas in the occipital pole and the thalamus.

(e) The right extrastriatal seed corresponded to a symmetric brain map known as the* visuospatial* ICN (Figure 6(e)) that included middle and inferior temporal gyri as well as visual association structures at the temporooccipital junction and extending laterally towards the primary visual cortex in the posterior and lateral occipital cortices. Moreover, this ICN map encompasses superior dorsal parietal regions and extra-primary areas of the visual cortex.

(f) The frontal eye fields (FEF) served as the seed region for the* dorsal attention *system (Figure 6(f)), displaying a pronounced symmetric activity in both hemispheres. This brain map encompasses the supplementary eye fields, small portions of the dorsolateral prefrontal cortex, the intraparietal cortices including the parietal eye fields, associative motor areas, and middle temporal structures encompassing visual associative structures. The cingulate gyrus extending from posterior towards anterior portions including the cingulate eye fields revealed also activity. Striatal regions exhibited strong iFC with the FEF; in detail, the putamen displayed the strongest connectivity while the caudate nucleus with adjusting thalamus was found to be less strongly functionally connected with the FEF. In summary, the dorsal attention ICN covers areas that are strongly associated with eye movement control.

(g) The* ventral attention *ICN (Figure 6(g)) has been computed by a basal ganglia seed in the right ventral striatum. Its iFC map covers large parts of the limbic system including the nucleus accumbens, the temporoparietal junction, and ventromedial prefrontal cortex.

(h) A second striatal seed within the caudate nucleus demonstrated strong activity in the basal ganglia and thalamus and is thus defined as the* basal ganglia thalamic *ICN (Figure 6(h)). The spatial pattern indicated weaker iFC with bilateral cerebellar regions and the right dorsolateral prefrontal cortex. Thus, the cortical activations were mainly found in the right hemisphere, with an overlap with the right frontoparietal control network.

(i) Placing a seed within the midbrain resulted in the* brainstem* ICN (Figure 6(i)) that included the brainstem extending from mesencephalic areas towards the medulla oblongata. The brainstem associated brain map encompasses bilateral thalamic areas. This resulting brain map is a mirror image with respect to the midline.

(j) The* cerebellar *ICN (Figure 6(j)) was identified by placing a seed in the right cerebellum. Weaker activations included middle temporal areas and bilateral thalamus.

**Figure 7:**
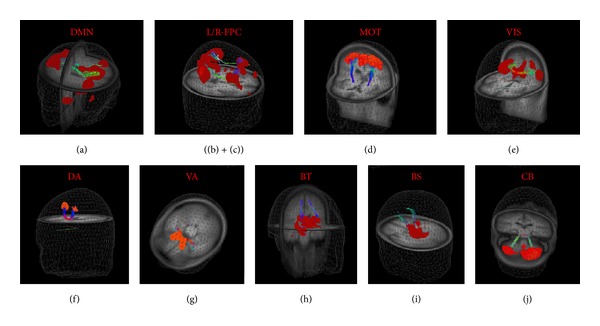
3D representations on the averaged MPRAGE template of ten ICNs ((a)–(j)) in MNI space with corresponding fiber tracts (FTs). (a) The default mode ICN (DMN) and cingulum bundle. ((b), (c)) The left and right frontoparietal control (FPC) ICNs and inferior longitudinal fasciculus. (d) The motor (MOT) ICN and corticospinal tracts. (e) The visuospatial (VIS) ICN and optic radiation. (f) The dorsal attention (DA) system and callosal radiation originating from callosal segment II. (g) The ventral attention (VA) ICN and callosal radiation originating from callosal segment I. (h) The basal ganglia thalamic (BT) ICN and thalamic radiation. (i) The brainstem (BS) ICN and corticopontine pathway. (j) The cerebellar (CB) ICN and superior cerebellar peduncle. Color coding is for visualization only.

The resulting ICNs in the elderly subjects (see Figure 6 and Supplementary Figure available online at http://dx.doi.org/10.1155/2014/947252) show a similar spatial distribution of the brain maps as compared to ICNs of younger subjects. These ICNs have been previously identified, that is, the “task-negative” DMN [12, 15], as well as the “task-positive” ICNs, comprising left and right lateralized frontoparietal control [49, 50], visuospatial [8, 20], motor [2, 52, 68] dorsal attention [4, 49], ventral attention [53, 69], basal ganglia thalamic [53, 69, 70], brainstem [49, 50], and cerebellar [49, 50]. ICNs capture fundamental units of functional organization [7]; for a detailed synopsis see also Table 2. Notably, we did not observe significant anticorrelated regions in any of the identified ICNs at group level and on individual basis. At group level, this finding was consistent for both data postprocessing approaches, that is, (i) arithmetically averaging of the individual brain maps and (ii) applying a one-sample *t*-test (including multiple comparison correction). The overall results of the investigated brain connectivity are illustrated in Figure 7 that shows the combination of the ten ICNs (Figure 6) with their corresponding DTI-based FTs: the DMN and the cingulum bundle [23], the left and right frontoparietal control ICNs and the inferior longitudinal fasciculus [61], the motor ICN and the corticospinal tracts [62, 63], the visuospatial ICN and the optic radiation [62, 63], the dorsal attention system and the callosal radiation originating from callosal segment II [63, 64], the ventral attention ICN and the callosal radiation originating from callosal segment I [63, 64], the basal ganglia thalamic ICN and thalamic radiation [65], the brainstem ICN and the corticopontine pathway [62, 66], and the cerebellar ICN and the superior cerebellar peduncle [67].

## 4. Discussion

### 4.1. Methodological Approach

- A framework has been presented that allows for iFC analysis and ICN identification by a five-item preprocessing followed by a three-step seed-based correlation analysis in order to obtain ICNs. The following algorithm implementations in the TIFT software [31] were adapted from DTI analysis and also applied for iFCMRI processing; data were resampled to a cubic 1 mm grid for further (complementary) analysis.
- Spatial Gaussian filtering was applied in order to optimize the sensitivity and specificity.
- Stereotaxic normalization to study-specific templates was performed both to an EPI-template for iFC and to (*b* = 0)- and FA-templates for DTI, respectively.

The parallels and cross-links to DTI analysis algorithms in a common software environment allow for further complementary iFC/DTI analysis at the group level targeting group comparisons [10].

### 4.2. Novelty of the Study

The novelty of this study comprises the following aspects.

- ICN identification in a sample of elderly subjects which is in the age range of many studies addressing neurodegenerative diseases. This is all the more important since it could be shown that age-dependent changes of the cerebral vasculature exist which may alter the neuronal-vascular coupling and thus the BOLD signal (in task-based fMRI investigations) [71].
- One prerequisite of the seed-voxel approach for ICN identification is an upsampling to a cubic 1 mm grid. Compared with ROI-seed-based approaches [10, 16, 45], the presently applied seed-voxel approach is supposed to provide some advantages: first, spatial smoothing with sufficient kernel size improves highly correlated time courses of adjacent voxels [45] so that the location of the seed-voxel is assumed to be robust against displacements. Second, although the data were stereotaxically normalized in a two-step procedure, small discrepancies in normalization are a common but confounding side effect due to the slightly different individual's brain anatomy. In particular, for small anatomical ROIs, a larger seed radius may exceed the true ROI by encompassing structures outside. Instead, selecting a single voxel in a 1 mm cubic grid on the basis of the averaged high-resolution image (e.g. MPRAGE) [46] may overcome this problem. In order to emphasize this statement, Figure 4 illustratively exemplifies that the PCC seed is less vulnerable against increasing spherical seed radii compared with the smaller caudate seed.
- Although the ICN identification at the group level directly reflects reliable ICNs with excellent signal-to-noise ratio compared to single subject ICNs, single subject ICNs showed sufficient quality for further comprehensive analysis at single subject level.
- IFCMRI analysis algorithms were included in a well-established software package which allows for easy complementary iFCMRI/DTI analysis. That way, prerequisites for combined functional and structural network analysis at the group level in studies of, for example, neurodegenerative diseases, are prepared.
- The eight-step proposed approach did not include any kind of brain masking (e.g., white matter mask or cerebrospinal fluid mask). No nuisance covariates such as whole brain signal, cerebrospinal fluid, white matter signals, or head motion parameters were regressed out, as addressed in the following.

### 4.3. Nuisance Covariates

In order to correct for nonneuronal BOLD signals [72], removing the effect of nuisance covariates is a common iFCMRI preprocessing step [47]. However, it remains unclear where the respective data for regressing out those covariates should be extracted [44]. In this study, no nuisance covariates were regressed out because the demonstrated ICNs in the elderly were unambiguously identified utilizing the proposed approach. The observed brain maps were remarkably similar compared to previous studies (e.g., [8, 20, 73]). The effect of regressing out movement, ventricle, and white matter covariates appears to be of minor impact which is in agreement with others [4, 74] who systematically investigated and illustrated these effects. However, those authors pointed out the strong influence of utilizing global signal regression that in turn induces the ongoing debated anticorrelated regions [5].

### 4.4. Anticorrelated Regions

The identified brain maps did not reveal anticorrelated regions, probably because we did not apply global mean regression in the data processing procedure. The commonly applied global mean regression is thought to induce anticorrelated regions [75] and is therefore still controversial in iFCMRI literature [44]. Hence, anticorrelated regions and their possible physiological interpretation are a matter of an ongoing debate [5, 76].

### 4.5. Prospects to Studies at the Group Level

While DTI-based comparisons at the group level require spatial smoothing (preferably by means of a Gaussian kernel with FWHM in the range of 6 mm to 12 mm or twice the scanner resolution [40]), statistical analysis of the ICNs can be directly applied to the iFCMRI-*z*(*r*) maps of subjects' data by performing a two-sided parametric Student's *t*-test for unequal variances [77] in order to contrast voxelwise differences groups. The resulting *P* values have to be corrected for multiple comparisons (e.g., by utilizing the false discovery rate (FDR) [78]), followed by correction for multiple comparisons at cluster level [79].

The step-by-step procedure presented in this study is an approach to implement complementary iFCMRI/DTI analysis at the group level (healthy controls' data as used in this study). The extension to comparisons of subject groups (consisting, e.g., of a patient and a control sample) could easily be performed. The complementary analysis in one single software environment allows for mapping structural damage (DTI metrics differences) in combination to detection of tract connections (DTI-based FT reconstructions) with functional alterations (hyper- or hypoconnectivity) of the corresponding networks.

The association between functional and structural connectivity in the brain at “rest” has been demonstrated for the DMN [10, 25]. More generally, functional components have been found to correspond to structural components for several portions of the brain [80]. This is one of the main goals of the human connectome project that aims at characterizing the brain function on the basis of functional and structural connectivity [81, 82]. The impact of both functional and structural components might be also important for the understanding of pathological conditions such as dementia [83]. In addition, the iFCMRI approach appears to be sensitive to characterize potential compensatory mechanisms [84]. Together, mapping the functional integrity of the human brain in neurological or psychiatric conditions emerges as a noninvasive sensitive approach to detect alterations in neuronal signaling.

### 4.6. Limitations

The healthy elderly participating in the present study were found to be free of any cognitive deficits; however, with respect to the years of education they were higher educated than the average adult population and may therefore be biased towards a somewhat higher cognitive reserve [85]. The limited number of included subjects (*N* = 12) can generally be considered as a limiting factor on the one hand. However, the low sample size might be of advantage for this specific investigation on the other hand because one aim was to identify consistent ICNs in a small number of cognitively sufficient characterized healthy adults.

With respect to data acquisition, the isometric recording resolution was 3 mm for iFCMRI and 2 mm for DTI, respectively; this limitation of different resolutions has been partially overcome by resampling to a 1 mm cubic grid. The DTI quality was further constrained by the minimum required number of gradients (i.e., *N* = 30) used for the structural analysis path. The ICNs comprised portions of the brain that may be coupled via complex fiber organizations. In order to track those axonal bundles and possibly cross-fibers, a higher number of gradient directions enable performing more subtle fiber tracking in optimized quality [31]. In addition, iFCMRI temporal resolution was typically low (TR = 2.2 s) which resulted, according to the sampling theorem, in aliasing effects caused by confounding frequency components *f* > 13.7/min (corresponding to TR) such as cardiac or respiratory oscillations [6].

## 5. Conclusion

In this study, it has been shown that it is possible to compute ICNs at single subject level as well as at the group level by use of a straightforward standardized procedure. Robust ICNs at the group level could be defined for a comparatively small number of contributing subjects (*N* = 12). With the parallel analysis approach of iFC and DTI in a single software environment (TIFT), it was shown that comprehensive analyses between functional network mapping (as assessed by iFCMRI analysis at the group level) and structural network mapping (as assessed by DTI-based FT of corresponding network tract systems) could be performed. The findings demonstrated here provide a methodological framework for future investigations aiming at contrasting pathological (neurodegenerative) conditions with healthy controls on the basis of multiparametric brain connectivity mapping.

## Supplementary Material

Supplementary Figure illustrates the more complete images of the 10 investigated brain maps according to Figure 6.

## Figures and Tables

**Table 1:** Demographic data of the subjects.

Parameter	Healthy elderly subjects
Number	12
Gender, M/F	7/5
Age/y	67.8 ± 6.8 (59.1–81.4)
MMSE	29.8 ± 0.5 (29.0–30.0)
DemTect	17 (10–13)
Years of education	12.5 (10.0–13.0)

Data are given as mean ± std (min–max).

MMSE: mini-mental state examination; DemTect [34].

**Table 2:** Definitions of seed-voxel location (1.0 × 1.0 × 1.0 mm^3^) in the MNI stereotaxic space with their corresponding intrinsic connectivity network (ICN). Given references provide the acknowledgement for the defined ICNs.

#	ICN	Seed-voxel			Seed-voxel region	Reference
		*X*	*Y*	*Z*		
A	Default mode network (DMN)	0	−55	26	Posterior cingulate cortex	Raichle et al., 2001 [15]; Greicius et al., 2003 [13]; Buckner et al., 2008 [12]
B	Left frontoparietal control (L-FPC)	−50	−52	49	Left Inferior parietal lobule	Vincent et al., 2008 [49]; Spoormaker et al., 2012 [50]; Beckmann et al., 2005 [19]
C	Right frontoparietal control (R-FPC)	50	−54	49	Right inferior parietal lobule	
D	Motor (MOT)	−27	−27	68	Motor cortex	Biswal et al., 1995 [2]; Wu, et al., 2009 [51]; Wu et al., 2011 [52]
E	Visuospatial (VIS)	47	−72	15	Extrastriate cortex	Smith et al., 2009 [8]; Laird et al., 2011 [20]; Beckmann et al., 2005 [19]
F	Dorsal attention (DA)	30	−9	54	Frontal eye field	Vincent et al., 2008 [49]; van Dijk et al., 2010 [4]
G	Ventral attention (VA)	11	13	0	Ventral striatum	Di Martino et al., 2008 [53]; Hacker et al., 2012 [46]
H	Basal ganglia thalamic (BT)	18	2	20	Caudate nucleus	Di Martino et al., 2008 [53]; Laird et al., 2011 [20]
I	Brainstem (BS)	2	−31	−20	Midbrain	Laird et al., 2011 [20]; Hacker et al., 2012 [46]
J	Cerebellum (CB)	32	−79	−34	Cerebellum	Smith et al., 2009 [8]; Laird et al., 2011 [20]

**Table 3:** Diffusion tensor imaging analysis: fiber tracking (FT) seeds in the MNI stereotaxic space corresponding to the intrinsic connectivity networks (ICNs).

#	ICN	FT seed			FT
		*X*	*Y*	*Z*	
A	Default mode network (DMN)	±8	−16	38	Cingulum bundles [23]
B, C	Left frontoparietal control (L-FPC)	±27	−40	53	Inferior longitudinal fasciculi [61]
D	Motor (MOT)	±25	−14	21	Corticospinal tracts [62, 63]
E	Visuospatial (VIS)	0	−14	21	Optic radiation [62, 63]
F	Dorsal attention (DA)	0	1	25	Callosal radiation originating from callosal segment II [63, 64]
G	Ventral attention (VA)	±18	−6	−8	Callosal radiation originating from callosal segment I [63, 64]
H	Basal ganglia thalamic (BT)	±25	−10	28	Thalamic radiation [65]
I	Brainstem (BS)	±4	−28	−13	Corticopontine pathway [62, 66]
J	Cerebellum (CB)	±15	−38	−29	Superior cerebellar peduncle [67]
