# Supplementary material for: Intrinsic Functional Connectivity Networks in Healthy Elderly Subjects: A Multiparametric Approach with Structural Connectivity Analysis
Source: Biomed Res Int. 2014 May 29;2014:947252. doi: 10.1155/2014/947252 (PMC4058120; doi:10.1155/2014/947252)
Supplement: Supplementary file 1 — Supplementary Figure illustrates the more complete images of the 10 investigated brain maps according to Figure 6. [file 947252.f1.pdf]

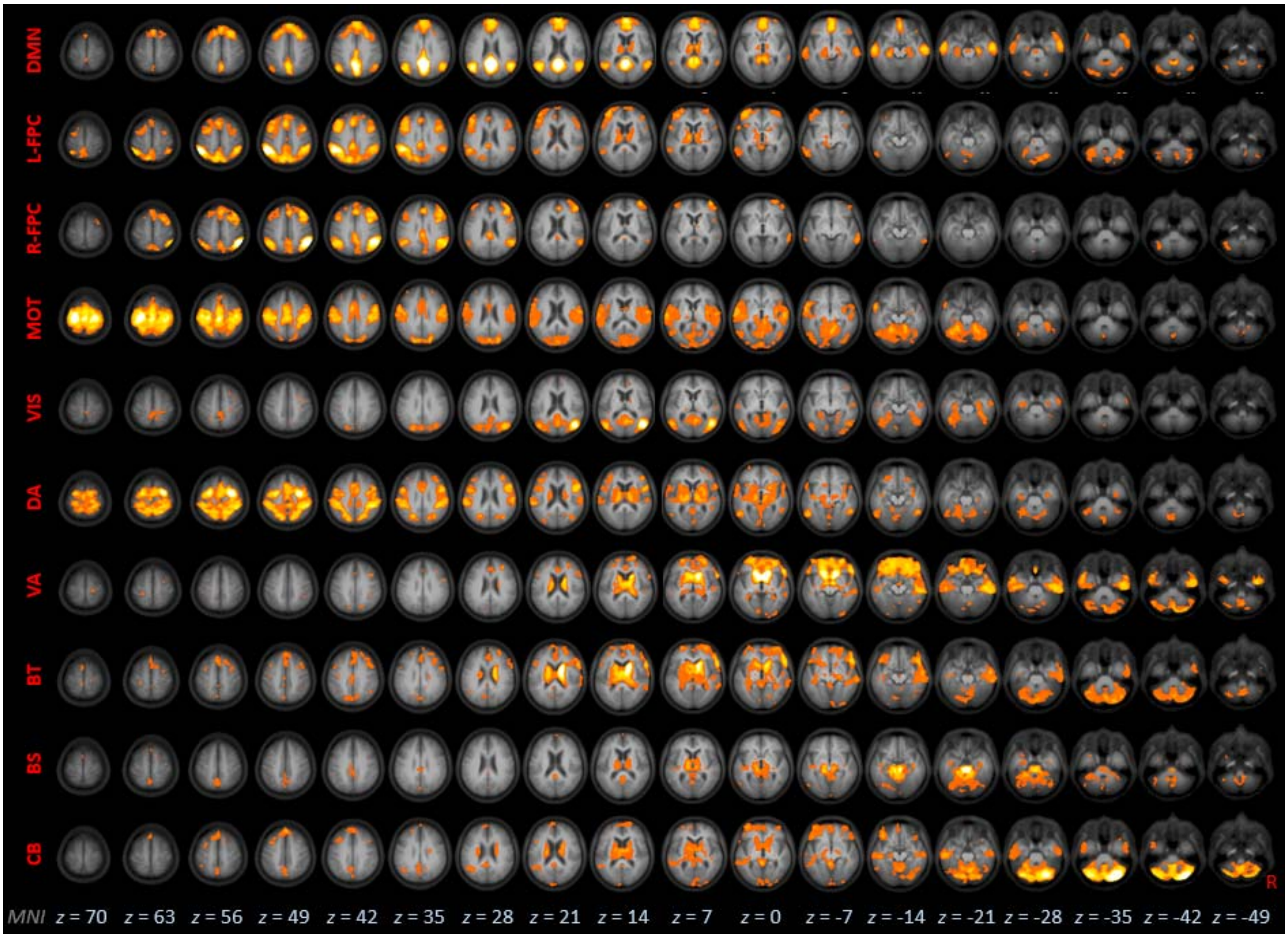

Supplementary Figure: Complementary axial slices of the ten investigated ICNs at the group level according to **Figure 6**. Abbreviations: DMN – Default Mode Network, PFC – Frontoparietal Control, MOT – Motor, VIS – Visuospatial, DA – Dorsal Attention, VA – Ventral Attention, BT – Basal ganglia Thalamic, BS – Brainstem, CB – Cerebellar.
